# Health Tract, No. 57

**Published:** 1865

**Authors:** 


### HEALTH TRACT, No 57.

### NURSING HINTS.

To the nurse is intrusted a holy human life, and to fail of duty by inattention or ignorance, is cruelly criminal.

1. The nurse should not eat, drink, or sleep in a sick-room.

2. Nor fast longer than five hours, whether a day or night watcher.

3. Always go into the room for day or night duty, with a full meal.

4. A strong body and a wide-awake mind are equally essential to a capable and efficient nurse; hence seven hours of consecutive sleep out of each twenty-four is a necessity.

5. Do not sit between the bed and the fire, or on the other side of the patient from an open door or window.

6. Clean your teeth, dress your hair, and wash your whole body well with soap and water after watching, so that you may sleep in clean linen, in no garment worn during your watching.

7. Wear as few woolen or dark clothes as possible; they hide dirt and harbor noxious exhalations.

8. Never speak in a whisper or under-tone in the sick-room, unless the patient is asleep; it engenders suspicions.

9. Avoid all discomposure, flurry, and noise, especially sudden, harsh, or discordant, and wear no creaking shoes or rustling garments.

10. Maintain at all times a countenance which is at once composed, self-possessed, cheerful, hopeful, kindly, confident, and sympathetic, else you are utterly unfit for the place

11. As far as possible, anticipate every want, without at the same time being officious. Avoid all unnecessary questionings, and do not be forever fixing things about the bed.

12. Keep scrupulously out of sight every thing in the shape of druggery, such as bottles, vials, spoons, pill-boxes, etc.

13. Do not allow any liquid thing to remain in the room one single moment longer than it is in use, not even a glass of ice-water.

14. Have no hanging garments in the sick-chamber, and as little woolen carpeting and bed-coverings as possible, and no bed or window-curtains.

15. Keep the room in perfect order, and arrange things with an eye to taste, neatness and cheerfulness.

16. If visitors are admitted, ask them to leave the room the moment conversation flags No patient can possibly desire to be gaped at in silence.

17. Never allow a frown, or an angry word, or an impatient expression of countenance, whatever may be the provocation. However "cross" the patient is, it is your business to be propitiative.

18. Guard against drafts of air and damp bed-clothes or garments.

19. Always have the fire-place open, and a window or door, as nearly opposite as possible, a little raised and lowered, or ajar. If there is no fire, have a lamp or candle burning in the fire-place, to create a draft up the chimney.

20. Let the room be as clean and as sunshiny as possible.

21. If fire is needed in the chamber, a thermometer should hang about five feet from the floor, opposite the fire-place, and should range about sixty degrees.

22. Let all kinds of impressive intelligence be communicated gradually, and as unimpressibly as possible.

23. Sleep is the best agency of recovery in all nature; hence never wake a sleeping patient, but promote sleep in all possible ways.

24. Do all you can to inspire confidence in the physician; never make a suggestion to him in the presence of the patient, and be faithful to his instructions.

